# Knowledge, Attitudes, and Practices Regarding Brucellosis Among the General Population in Qassim Region, Saudi Arabia: A Cross-Sectional Study

**DOI:** 10.7759/cureus.41461

**Published:** 2023-07-06

**Authors:** Ayman A Harbi, Abdulmalik S Almarshad, Omar A Alaqeel, Bilal S Al-Mushaigah, Abdullah I Aldekhail

**Affiliations:** 1 Department of Medicine, Qassim University, Buraydah, SAU

**Keywords:** zoonotic transmission, preventive practices, health education & awareness, knowledge, qassim region, saudi arabia, brucellosis

## Abstract

Background

Brucellosis is an endemic infection in the Middle East. The World Health Organization has identified the illness as one of the most prevalent "neglected zoonotic diseases." Public perceptions regarding brucellosis infection vary across different regions. Thus, this study aimed to assess the knowledge, attitudes, and practices concerning brucellosis among the general population in the Qassim region of Saudi Arabia.

Methods

A descriptive, cross-sectional study was conducted in the Qassim region of Saudi Arabia between March 2023 and May 2023. We distributed an online questionnaire through social media platforms and received a total of 1,244 responses from different governorates in the Qassim region.

Results

The level of awareness of brucellosis was moderate. Out of the 1,244 participants, 706 (56.8%) were aware of the disease. Among the participants who were aware of the disease, their knowledge of brucellosis was assessed. The majority of these respondents demonstrated a good level of knowledge (70.5%). Regarding attitudes and practices toward brucellosis, no significant association was found between the participants’ level of knowledge and their attitudes.

Conclusion

Awareness levels about brucellosis in the Qassim region vary depending on age group, area of residency, and other factors. The younger age group and certain governorates in the Qassim region showed low levels of awareness. Moreover, participants with good knowledge about the disease obtained their information from healthcare practitioners. The significant difference in our findings between Qassim’s governorates demonstrates the need for more targeted awareness campaigns in areas with lower levels of awareness.

## Introduction

Brucella species are facultative, intracellular, Gram-negative coccobacilli bacteria that cause brucellosis [1]. The Brucella species responsible for human brucellosis include B. melitensis, B. suis, B. abortus, and B. canis [2]. Brucellosis, which is also commonly known as "Malta fever" in Saudi Arabia, is a widespread zoonotic disease transmitted through contact with infected animals or consumption of contaminated animal products. Cattle, swine, goats, sheep, and dogs are common hosts for brucellosis [2,3]. The growth of animal industries, urbanization, and poor hygiene in animal husbandry and food handling all contribute to the ongoing threat of brucellosis to public health. However, human-to-human transmission remains relatively rare [4].

The incubation period of brucellosis typically ranges from two to four weeks, although it can vary between five days and six months. The initial clinical symptoms of brucellosis are nonspecific and include fever, sweating, chills, loss of appetite, headache, fatigue, and lethargy, as well as muscle, joint, and back pain. In addition, brucellosis causes multiple complications, with endocarditis being the principal cause of death. The most common and devastating complications in patients with brucellosis include arthritis, orchitis, spleen or liver inflammation, and central nervous system involvement [1,2].

For the diagnosis of brucellosis, diagnostic criteria are published by the Centers for Disease Control and Prevention and the Council of State and Territorial Epidemiologists. These criteria consider culture and identification of Brucella species from clinical specimens or a fourfold or greater rise in Brucella antibody titer between acute and convalescent-phase serum specimens obtained at least two weeks apart as definitive criteria [5]. However, due to a shortage of medical professionals capable of diagnosing this disease, brucellosis in humans is frequently misdiagnosed as various other illnesses or may remain undiagnosed [6].

The prevention of brucellosis is a complex process that involves identifying and mitigating risk factors. Unfortunately, vaccines are not available for humans. Therefore, the prevention of human infection primarily focuses on raising awareness about the disease and improving hygiene in occupational, food, and laboratory settings. In addition, one of the most effective ways to prevent human brucellosis is to eliminate animal infection, which can be done through animal vaccinations and other control measures [4].

In terms of brucellosis awareness in Saudi Arabia, a study conducted in Jazan involving 1,055 participants found that only 50% of the participants are aware of brucellosis. Approximately 70% of those who are aware have demonstrated good knowledge. The preventive practices undertaken by livestock owners against brucellosis vary from average to unsatisfactory. Interestingly, the study revealed that having good knowledge has no significant influence on their practices [7]. Similarly, a study conducted in the Aseer Region of Saudi Arabia with 311 participants showed that the majority have a fair knowledge and satisfactory attitudes and practices regarding brucellosis. However, instances of incorrect practices and misconceptions about the disease remain. The study emphasized the importance of public awareness campaigns to improve knowledge and promote safe practices [6].

In Namibia, a cross-sectional study assessed the knowledge, attitudes, and practices regarding brucellosis among cattle farmers, meat handlers, and medical professionals. The study found that the level of awareness of brucellosis is relatively low, with a 43.5% awareness level among participants, with medical professionals exhibiting the highest level of awareness. This highlights the need for improved education and awareness programs [8]. A study conducted in Kenya revealed that knowledge of brucellosis is generally low among 120 pastoralists. Only 79% have heard of brucellosis, and they have a limited understanding of its causes and prevention methods. The study emphasized the necessity of public awareness campaigns to enhance knowledge and promote safe practices among pastoralists in Kenya [9].

Furthermore, a meta-analysis examining brucellosis awareness and knowledge across 22 countries, primarily in Asia and Africa, reported that knowledge levels regarding the zoonotic nature, modes of transmission, and symptoms of brucellosis are lower than the overall level of awareness. The analysis concluded that people’s awareness and knowledge of brucellosis are insufficient [10].

Collectively, these studies and meta-analyses highlighted varying levels of awareness, knowledge, attitudes, and practices regarding brucellosis among the general population in different regions. They emphasized the importance of tailored public awareness campaigns, improved education, and the promotion of safe practices to effectively prevent and control brucellosis.

The Qassim region, located in Saudi Arabia, lacks studies that investigate awareness, knowledge, and practice regarding brucellosis. Therefore, this study aims to address this research gap by assessing the level of awareness, knowledge, and practice regarding brucellosis among the general population in the Qassim region of Saudi Arabia.

## Materials and methods

A descriptive, cross-sectional study using qualitative data was conducted in the Qassim region of Saudi Arabia from March 2023 to May 2023. Ethical approval for the study was obtained from the Qassim Region Research Ethics Committee. A convenience non-probability sampling technique was utilized, encompassing all Qassim governorates, including Buraidah, Unaizah, Ar Rass, Al Mithnab, Al Bukayriyah, Al Badayea, Asyah, Al Nabhaniyah, Uyun AlJiwa, Uglat Asugour, Dariyah, Riyadh Al Khabra, and Al Shimasiyah. The inclusion criteria comprised adults aged 18 years or older who reside in the Qassim region, whereas the exclusion criteria involved individuals under 18 years old or those not from the Qassim region.

The study involved 1,244 participants who willingly participated in the research. Data were collected using a validated questionnaire after obtaining consent from the original author [7]. The questionnaire was divided into three sections. The first section focused on sociodemographic data; the second section assessed knowledge regarding brucellosis; and the final section explored practices and attitudes toward the disease.

Statistical analysis

In the present study, knowledge regarding brucellosis was based on 12 items. Each correct response was assigned a value of one, whereas incorrect responses were assigned a value of zero. Therefore, the total knowledge score ranged from zero to 12. A percentage score was computed using the following formula: Percent score = (raw score × 100)/12. A good knowledge level was considered at a score of over 50% of the overall score.

Data analysis was performed using R version 4.2.2 (RStudio, Boston, MA). Categorical variables were expressed using frequencies and percentages, whereas continuous variables were presented using medians and interquartile ranges (IQRs). The factors associated with participants’ awareness and knowledge were assessed using Pearson’s chi-squared test or Fisher’s exact test, as applicable. Regression analysis was conducted to identify the independent predictors of awareness (no vs. yes) and knowledge (poor vs. good). Each variable was entered as a dependent variable in a separate binary logistic regression model. The independent variables included in the models were those that showed a statistically significant association with the dependent variable. The results of the regression model were expressed as odds ratios (ORs) with 95% confidence intervals (CIs). Statistical significance was considered when p < 0.05.

## Results

Sociodemographic characteristics

Initially, a total of 1297 responses were received. However, 53 respondents declined to participate. Accordingly, 1,244 responses were analyzed in the current study. More than half were female (51.5%), unmarried (55.6%), held a bachelor’s degree (57.2%), and aged between 18 and 30 years (58.3%). In addition, 49.9% of the participants had a monthly income of more than 10,000 SAR. More than one-third of the respondents were students (38.0%). The city of Buraydah had the highest representation among the participants, accounting for 43.5% of the sample. Notably, 59 respondents (4.7%) reported a previous brucellosis infection (Table 1).

**Table 1:** Sociodemographic characteristics

Parameters	Category	*N* (%)
Gender	Male	603 (48.5%)
	Female	641 (51.5%)
Age (year)	18–30	724 (58.3%)
	31–40	240 (19.3%)
	41–50	173 (13.9%)
	51–60	75 (6.0%)
	>60	29 (2.3%)
Occupation	Student	473 (38.0%)
	Not working	242 (19.5%)
	Working	429 (34.5%)
	Retired	100 (8.0%)
Marital status	Single/divorced/widowed	692 (55.6%)
	Married	552 (44.4%)
Residence area	Buraydah	541 (43.5%)
	Unaizah	169 (13.6%)
	Alrass	207 (16.6%)
	Bukayriyah	92 (7.4%)
	Badaya	68 (5.5%)
	Riyadh Al Khabra	103 (8.3%)
	Methnab	9 (0.7%)
	Uglat Asugour	6 (0.5%)
	Shamasiya	5 (0.4%)
	Asiyah	10 (0.8%)
	Nabhaniya	5 (0.4%)
	Dariya	8 (0.6%)
	Oyon Al-Jawaa	21 (1.7%)
Family size (number of individuals)	Not married	400 (32.2%)
	1–5	376 (30.2%)
	6–7	251 (20.2%)
	8–10	157 (12.6%)
	>10	60 (4.8%)
Educational level	Uneducated	9 (0.7%)
	Primary/middle/secondary	291 (23.4%)
	Diploma	152 (12.2%)
	Bachelor	712 (57.2%)
	Post-graduate	80 (6.4%)
Monthly income (SAR)	<5,000	267 (21.5%)
	5,000–10,000	356 (28.6%)
	>10,000	621 (49.9%)
Ever had brucellosis	Yes	59 (4.7%)

Level of awareness regarding brucellosis and its associated factors

**Figure 1:**
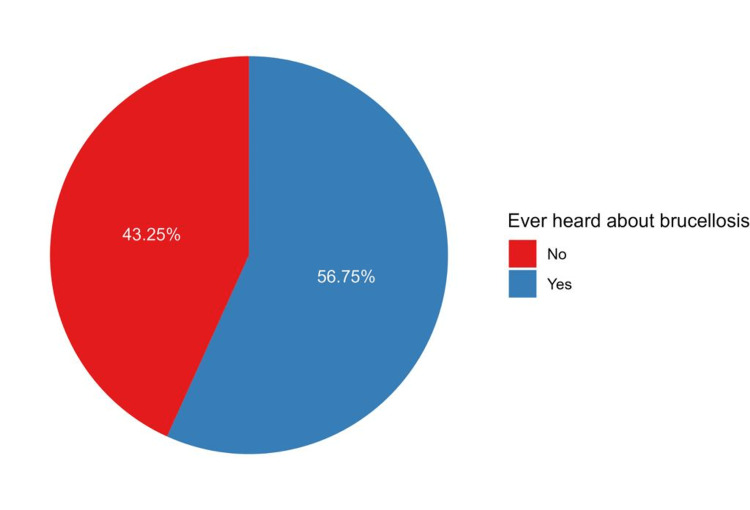
Pie chart showing the awareness levels of the study participants (n = 1244)

The level of awareness regarding brucellosis and its associated factors has been influenced by participants’ age, gender, marital status, job status, residence area, and education level. In our study, 706 participants (56.8%) were aware of brucellosis (Figure 1). The levels of awareness significantly differed based on the participants’ age (p < 0.001), occupational status (p < 0.001), marital status (p < 0.001), family size (p < 0.001), educational level (p < 0.001), monthly income (p < 0.001), history of brucellosis (p = 0.010), and residency in Oyon Al-Jawaa (p = 0.007) (Table 2).

**Table 2:** Factors associated with participants’ level of awareness regarding brucellosis

Parameters	Category	Aware about brucellosis		
		No, *N *= 538	Yes, *N* = 706	p-value
Gender	Male	255 (42.3%)	348 (57.7%)	0.508
	Female	283 (44.1%)	358 (55.9%)	
Age (year)	18–30	429 (59.3%)	295 (40.7%)	<0.001
	31–40	67 (27.9%)	173 (72.1%)	
	41–50	26 (15.0%)	147 (85.0%)	
	51–60	9 (12.0%)	66 (88.0%)	
	>60	6 (20.7%)	23 (79.3%)	
Occupation	Student	299 (63.2%)	174 (36.8%)	<0.001
	Not working	102 (42.1%)	140 (57.9%)	
	Working	116 (27.0%)	313 (73.0%)	
	Retired	21 (21.0%)	79 (79.0%)	
Marital status	Single/divorced/widowed	412 (59.5%)	280 (40.5%)	<0.001
	Married	126 (22.8%)	426 (77.2%)	
Buraydah	No	300 (42.7%)	403 (57.3%)	0.642
	Yes	238 (44.0%)	303 (56.0%)	
Unaizah	No	454 (42.2%)	621 (57.8%)	0.068
	Yes	84 (49.7%)	85 (50.3%)	
Alrass	No	452 (43.6%)	585 (56.4%)	0.588
	Yes	86 (41.5%)	121 (58.5%)	
Bukayriyah	No	499 (43.3%)	653 (56.7%)	0.863
	Yes	39 (42.4%)	53 (57.6%)	
Badaya	No	513 (43.6%)	663 (56.4%)	0.267
	Yes	25 (36.8%)	43 (63.2%)	
Riyadh Al Khabra	No	495 (43.4%)	646 (56.6%)	0.748
	Yes	43 (41.7%)	60 (58.3%)	
Methnab	No	533 (43.2%)	702 (56.8%)	0.512
	Yes	5 (55.6%)	4 (44.4%)	
Uglat Asugour	No	536 (43.3%)	702 (56.7%)	0.704
	Yes	2 (33.3%)	4 (66.7%)	
Shamasiya	No	537 (43.3%)	702 (56.7%)	0.397
	Yes	1 (20.0%)	4 (80.0%)	
Asiyah	No	532 (43.1%)	702 (56.9%)	0.344
	Yes	6 (60.0%)	4 (40.0%)	
Nabhaniya	No	536 (43.3%)	703 (56.7%)	>0.999
	Yes	2 (40.0%)	3 (60.0%)	
Dariya	No	534 (43.2%)	702 (56.8%)	0.733
	Yes	4 (50.0%)	4 (50.0%)	
Oyon Al-Jawaa	No	535 (43.7%)	688 (56.3%)	0.007
	Yes	3 (14.3%)	18 (85.7%)	
Family size (number of individuals)	Not married	238 (59.5%)	162 (40.5%)	<0.001
	1–5	121 (32.2%)	255 (67.8%)	
	6–7	75 (29.9%)	176 (70.1%)	
	8–10	74 (47.1%)	83 (52.9%)	
	>10	30 (50.0%)	30 (50.0%)	
Educational level	Uneducated	5 (55.6%)	4 (44.4%)	<0.001
	Primary/middle/secondary	164 (56.4%)	127 (43.6%)	
	Diploma	58 (38.2%)	94 (61.8%)	
	Bachelor	289 (40.6%)	423 (59.4%)	
	Post-graduate	22 (27.5%)	58 (72.5%)	
Monthly income (SAR)	<5,000	169 (63.3%)	98 (36.7%)	<0.001
	5,000–10,000	133 (37.4%)	223 (62.6%)	
	>10,000	236 (38.0%)	385 (62.0%)	
Have you ever had brucellosis?	No	522 (44.1%)	663 (55.9%)	0.01
	Yes	16 (27.1%)	43 (72.9%)	

The regression analysis revealed that the level of awareness regarding brucellosis was independently associated with age, from 31 to 40 years (p < 0.001), 41 to 50 years (p < 0.001), 51 to 60 years (p < 0.001), and >60 years (p = 0.003). In addition, individuals with a monthly income ranging from 5,000 to 10,000 SAR (p < 0.001) and over 10,000 SAR (p = 0.003) showed higher levels of awareness. Being married (p = 0.007) and residing in Oyon Al-Jawaa (p = 0.033) were also significant predictors of awareness (Table 3).

**Table 3:** Predictors of participants’ awareness regarding brucellosis

Parameters	Category	OR	95% CI	p-value
Age (year)	18–30	Ref	Ref	
	31–40	2.27	1.48, 3.48	<0.001
	41–50	5.03	2.83, 9.15	<0.001
	51–60	9.71	3.97, 26.1	<0.001
	>60	5.87	1.89, 20.2	0.003
Occupation	Student	Ref	Ref	
	Not working	1.05	0.70, 1.57	0.814
	Working	1.29	0.87, 1.91	0.211
	Retired	0.52	0.23, 1.19	0.121
Marital status	Single/divorced/widowed	Ref	Ref	
	Married	1.88	1.19, 2.97	0.007
Oyon Al-Jawaa	No	Ref	Ref	
	Yes	4.12	1.26, 18.7	0.033
Family size (number of individuals)	Not married	Ref	Ref	
	1–5	0.79	0.51, 1.20	0.269
	6–7	1.02	0.67, 1.55	0.930
	8–10	0.71	0.46, 1.11	0.136
	>10	0.82	0.44, 1.51	0.523
Educational level	Uneducated	Ref	Ref	
	Primary/middle/secondary	1.43	0.33, 6.68	0.631
	Diploma	1.78	0.40, 8.43	0.448
	Bachelor	2.43	0.57, 11.2	0.231
	Post-graduate	2.52	0.54, 12.6	0.241
Monthly income (SAR)	<5,000	Ref	Ref	
	5,000–10,000	2.03	1.41, 2.94	<0.001
	>10,000	1.68	1.19, 2.37	0.003
Have you ever had brucellosis?	No	Ref	Ref	
	Yes	1.75	0.92, 3.44	0.095

Knowledge regarding brucellosis and its associated factors

**Figure 2:**
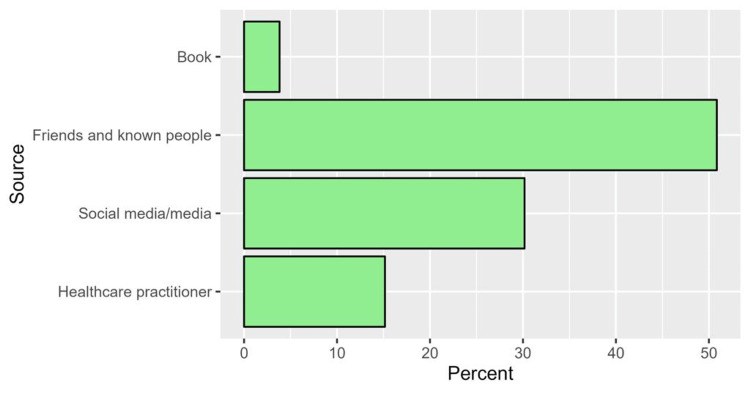
Percentages of sources of information regarding brucellosis

Regarding knowledge about brucellosis, participants were asked about the source of information about the disease, whether humans can get infected, the symptoms of the disease, and its mode of transmission. Among the participants who were aware of the disease (n = 706), the median IQR knowledge score was 8.0 (6.0 to 10.0), with a minimum score of zero and a maximum score of 12. The items assessing knowledge showed an acceptable level of internal consistency (Cronbach’s alpha = 0.741). In terms of the sources of information, the most frequently reported sources were friends/relatives (50.8%) and social media/media (30.2%) (Figure 2). Most participants believed that humans can contract brucellosis infection (83.9%) and that infected individuals can develop fever (74.4%) and arthritis (63.5%). Regarding the transmission of brucellosis, most participants believed that brucellosis infection can be transmitted from an infected animal to a healthy person (77.6%) and from an infected animal to a healthy animal (69.5%). However, only 47.6% of participants stated that the infection could be transmitted from an infected person to a healthy person. The detailed responses to the knowledge-related questions, along with the correct answers, are provided in Table 4.

**Table 4:** Participants’ responses to the knowledge items *An asterisk indicates a correct answer

Item	Response	*N *(%)
What animal species can become infected?	I do not know	143 (20.3%)
	Sheep, cattle*	438 (62.0%)
	Camel*	9 (1.3%)
	All mammals*	116 (16.4%)
Can a person get infected?	I do not know	64 (9.1%)
	No	50 (7.1%)
	Yes*	592 (83.9%)
Can fever be a symptom caused by brucellosis in humans?	I do not know	148 (21.0%)
	No	33 (4.7%)
	Yes*	525 (74.4%)
Can arthritis be a symptom caused by brucellosis in humans?	I do not know	225 (31.9%)
	No	33 (4.7%)
	Yes*	448 (63.5%)
Can skin problems be a symptom caused by brucellosis in humans?	I do not know	323 (45.8%)
	No	132 (18.7%)
	Yes*	251 (35.6%)
Are there any vaccinations for animals against brucellosis?	I do not know	276 (39.1%)
	No	37 (5.2%)
	Yes*	393 (55.7%)
Infection can be transmitted from an infected animal to a healthy animal	I do not know	180 (25.5%)
	True*	491 (69.5%)
	False	35 (5.0%)
The infection can be transmitted from an infected animal to a healthy person	I do not know	119 (16.9%)
	True*	548 (77.6%)
	False	39 (5.5%)
The infection can be transmitted from an infected person to a healthy person	I do not know	217 (30.7%)
	True*	336 (47.6%)
	False	153 (21.7%)
*Brucella *bacteria can cause serious illness or even death	I do not know	326 (46.2%)
	True*	298 (42.2%)
	False	82 (11.6%)
This disease in humans can be treated with medication	I do not know	115 (16.3%)
	True*	564 (79.9%)
	False	27 (3.8%)
All bacteria can be killed when milk is boiled/pasteurized to at least 63°C	I do not know	210 (29.7%)
	True*	429 (60.8%)
	False	67 (9.5%)

**Figure 3:**
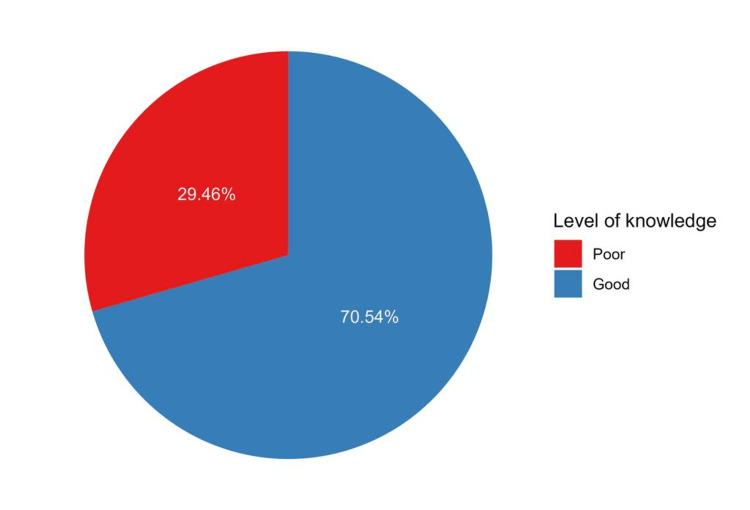
Pie chart showing the levels of knowledge of the participants who were aware of brucellosis (n = 706)

More than 50% of the questions were correctly answered by 498 participants out of 702 (70.5%) (Figure 3), indicating a good level of knowledge. This good knowledge was significantly associated with the participants’ occupation, monthly income, and source of knowledge (p ≤ 0.05). Significant differences in knowledge were noted based on residence in Buraydah (p < 0.001), Badaya (p = 0.004), and Riyadh Al Khabra (p = 0.014) (Table 5). However, participants who relied on healthcare practitioners as their source of information were more likely to possess good knowledge regarding brucellosis (p = 0.020). Furthermore, participants residing in Buraydah were more likely to be knowledgeable (p = 0.036), whereas those residing in Badaya (p = 0.007) and Riyadh Al Khabra (p = 0.029) were less likely to have good knowledge (Table 6).

**Table 5:** Factors associated with participants’ knowledge of brucellosis

Parameters	Category	Knowledge level		
		Poor, *N* = 208	Good, *N *= 498	p-value
Gender	Male	102 (29.3%)	246 (70.7%)	0.931
	Female	106 (29.6%)	252 (70.4%)	
Age (year)	18–30	98 (33.2%)	197 (66.8%)	0.192
	31–40	47 (27.2%)	126 (72.8%)	
	41–50	36 (24.5%)	111 (75.5%)	
	51–60	16 (24.2%)	50 (75.8%)	
	>60	9 (39.1%)	14 (60.9%)	
Occupation	Student	54 (31.0%)	120 (69.0%)	0.007
	Not working	56 (40.0%)	84 (60.0%)	
	Working	75 (24.0%)	238 (76.0%)	
	Retired	23 (29.1%)	56 (70.9%)	
Marital status	Single/divorced/widowed	92 (32.9%)	188 (67.1%)	0.109
	Married	116 (27.2%)	310 (72.8%)	
Buraydah	No	141 (35.0%)	262 (65.0%)	<0.001
	Yes	67 (22.1%)	236 (77.9%)	
Unaizah	No	184 (29.6%)	437 (70.4%)	0.791
	Yes	24 (28.2%)	61 (71.8%)	
Alrass	No	168 (28.7%)	417 (71.3%)	0.34
	Yes	40 (33.1%)	81 (66.9%)	
Bukayriyah	No	189 (28.9%)	464 (71.1%)	0.289
	Yes	19 (35.8%)	34 (64.2%)	
Badaya	No	187 (28.2%)	476 (71.8%)	0.004
	Yes	21 (48.8%)	22 (51.2%)	
Riyadh Al Khabra	No	182 (28.2%)	464 (71.8%)	0.014
	Yes	26 (43.3%)	34 (56.7%)	
Methnab	No	207 (29.5%)	495 (70.5%)	>0.999
	Yes	1 (25.0%)	3 (75.0%)	
Uglat Asugour	No	205 (29.2%)	497 (70.8%)	0.079
	Yes	3 (75.0%)	1 (25.0%)	
Shamasiya	No	207 (29.5%)	495 (70.5%)	>0.999
	Yes	1 (25.0%)	3 (75.0%)	
Asiyah	No	207 (29.5%)	495 (70.5%)	>0.999
	Yes	1 (25.0%)	3 (75.0%)	
Nabhaniya	No	208 (29.6%)	495 (70.4%)	0.559
	Yes	0 (0.0%)	3 (100.0%)	
Dariya	No	206 (29.3%)	496 (70.7%)	0.585
	Yes	2 (50.0%)	2 (50.0%)	
Oyon Al-Jawaa	No	205 (29.8%)	483 (70.2%)	0.228
	Yes	3 (16.7%)	15 (83.3%)	
Family size (number of individuals)	Not married	50 (30.9%)	112 (69.1%)	0.762
	1 to 5	77 (30.2%)	178 (69.8%)	
	6–7	45 (25.6%)	131 (74.4%)	
	8–10	26 (31.3%)	57 (68.7%)	
	>10	10 (33.3%)	20 (66.7%)	
Educational level	Uneducated	1 (25.0%)	3 (75.0%)	0.165
	Primary/middle/secondary	40 (31.5%)	87 (68.5%)	
	Diploma	35 (37.2%)	59 (62.8%)	
	Bachelor	121 (28.6%)	302 (71.4%)	
	Post-graduate	11 (19.0%)	47 (81.0%)	
Monthly income (SAR)	<5,000	36 (36.7%)	62 (63.3%)	0.014
	5,000–10,000	76 (34.1%)	147 (65.9%)	
	>10,000	96 (24.9%)	289 (75.1%)	
Ever had brucellosis	No	193 (29.1%)	470 (70.9%)	0.421
	Yes	15 (34.9%)	28 (65.1%)	
Sources of your knowledge	Healthcare practitioner	19 (17.8%)	88 (82.2%)	0.023
	Social media/media	69 (32.4%)	144 (67.6%)	
	Friends and known people	114 (31.8%)	245 (68.2%)	
	Book	6 (22.2%)	21 (77.8%)	

**Table 6:** Predictors of good knowledge among participants who were aware about brucellosis

Parameters	Category	OR	95% CI	p-value
Occupation	Student	Ref	Ref	
	Not working	0.75	0.46, 1.22	0.249
	Working	1.42	0.91, 2.21	0.122
	Retired	1.08	0.59, 2.03	0.806
Buraydah	No	Ref	Ref	
	Yes	1.49	1.03, 2.18	0.036
Badaya	No	Ref	Ref	
	Yes	0.40	0.20, 0.78	0.007
Riyadh Al Khabra	No	Ref	Ref	
	Yes	0.52	0.29, 0.94	0.029
Monthly income (SAR)	<5,000	Ref	Ref	
	5,000–10,000	1.07	0.63, 1.80	0.795
	>10,000	1.49	0.89, 2.47	0.127
Sources of your knowledge of brucellosis	Friends and known people	Ref	Ref	
	Healthcare practitioner	1.96	1.13, 3.52	0.020
	Social media/media	0.96	0.66, 1.40	0.825
	Book	1.45	0.59, 4.13	0.446

Attitudes and practices toward brucellosis

Participants’ attitudes and practices toward brucellosis were evaluated based on the following items: meat consumption, slaughtering practice, wearing gloves, and participation in delivering pregnant animals (Table 7). In our study, 75 participants declared that they owned or raised livestock. Among them, 30.7% always consumed meat from the animals that they raised and nurtured, 20.0% always performed animal slaughter, 6.7% always used gloves when caring for the animal, 6.7% assisted in delivering pregnant animals with an abortion, 18.7% consumed fresh milk without boiling it, and 12.0% believed that homemade cheese was tastier than store-bought cheese in sealed packaging. Furthermore, approximately two-thirds of the participants who were aware (65.3%) had vaccinated their livestock against brucellosis. No significant differences were found in the attitudes and practices toward brucellosis between participants with and without good knowledge levels.

**Table 7:** Patterns of attitudes and practices toward brucellosis and its associated factors among participants who own or raise cattle (n = 75)

Parameters	Category	Overall, N= 75	Knowledge level		
			Poor, *N* = 26	Good, N = 49	p-value
I eat meat from what I raise and nurture	Does not apply	6 (8.0%)	3 (50.0%)	3 (50.0%)	0.255
	Never	15 (20.0%)	8 (53.3%)	7 (46.7%)	
	Sometimes	31 (41.3%)	9 (29.0%)	22 (71.0%)	
	Always	23 (30.7%)	6 (26.1%)	17 (73.9%)	
I myself slaughter the livestock I own/keep	Does not apply	13 (17.3%)	6 (46.2%)	7 (53.8%)	0.739
	Never	13 (17.3%)	5 (38.5%)	8 (61.5%)	
	Sometimes	34 (45.3%)	10 (29.4%)	24 (70.6%)	
	Always	15 (20.0%)	5 (33.3%)	10 (66.7%)	
I use gloves when I take care of my livestock	Does not apply	21 (28.0%)	9 (42.9%)	12 (57.1%)	0.148
	Never	23 (30.7%)	9 (39.1%)	14 (60.9%)	
	Sometimes	26 (34.7%)	5 (19.2%)	21 (80.8%)	
	Always	5 (6.7%)	3 (60.0%)	2 (40.0%)	
I help deliver a pregnant animal with an abortion	Does not apply	21 (28.0%)	9 (42.9%)	12 (57.1%)	0.767
	Never	25 (33.3%)	8 (32.0%)	17 (68.0%)	
	Sometimes	24 (32.0%)	7 (29.2%)	17 (70.8%)	
	Always	5 (6.7%)	2 (40.0%)	3 (60.0%)	
In my opinion, homemade cheese is tastier than purchased cheese in sealed packaging certified by the Ministry of Health	Does not apply	18 (24.0%)	7 (38.9%)	11 (61.1%)	0.664
	Never	21 (28.0%)	5 (23.8%)	16 (76.2%)	
	Sometimes	27 (36.0%)	11 (40.7%)	16 (59.3%)	
	Always	9 (12.0%)	3 (33.3%)	6 (66.7%)	
I drink fresh milk without boiling it	Does not apply	14 (18.7%)	8 (57.1%)	6 (42.9%)	0.062
	Never	24 (32.0%)	5 (20.8%)	19 (79.2%)	
	Sometimes	23 (30.7%)	6 (26.1%)	17 (73.9%)	
	Always	14 (18.7%)	7 (50.0%)	7 (50.0%)	
Ever vaccinated the livestock against brucellosis	No	26 (34.7%)	10 (38.5%)	16 (61.5%)	0.615
	Yes	49 (65.3%)	16 (32.7%)	33 (67.3%)	

## Discussion

Awareness regarding brucellosis

The findings of this study provide insights into the level of awareness regarding brucellosis in the Qassim region of Saudi Arabia. The study revealed that, among the participants, 706 (56.8%) had heard about brucellosis, indicating a moderate level of awareness within the population. These results are consistent with a systematic review and meta-analysis of observational studies that reported a pooled awareness level of 55.5% across different regions worldwide, including Asia, Africa, Europe, South/Central America, and North America [10]. A similar awareness level (50%) was found in a study conducted in Jazan [7]. However, a higher level of awareness (73.6%) was reported in Asser, which focused on an older population [6].

This study also explored the factors influencing awareness of brucellosis and identified several significant associations. Age emerged as a significant predictor of awareness, with participants aged 31 to 40 years, 41 to 50 years, 51 to 60 years, and over 60 years demonstrating higher levels of awareness. This finding is consistent with other studies conducted in different regions, suggesting that older age groups tend to have a better understanding of brucellosis. This finding indicates the importance of targeted awareness campaigns and educational initiatives in enhancing the knowledge and understanding of brucellosis among younger age groups [6,7].​​​​​

Marital status also emerged as a significant predictor of awareness, with married individuals showing higher levels of awareness compared to other social statuses. These findings mirror the results of a study in Jazan, where being married was associated with better awareness and knowledge of the disease [7]. Educational level and monthly income were identified as important factors associated with awareness. Higher educational levels and income were consistently linked to increased awareness of brucellosis in this study and in other studies [6,7,11]. These findings suggest that socioeconomic factors play a role in shaping knowledge and awareness levels regarding brucellosis.

Notably, a study conducted in Jordan reported 100% awareness of brucellosis among the surveyed farms, indicating a high level of awareness within the farming community in Jordan [12]. This finding underscores the influence of occupation and exposure to animal health issues on awareness levels. However, interestingly, a study conducted in urban and peri-urban areas of Tajikistan found that the majority (85%) of farmers had never heard of brucellosis [11]. This finding highlights the idea that the level of awareness of brucellosis can vary significantly across different regions and populations, as demonstrated by the differences in awareness levels reported in various studies. Understanding these differences is crucial for designing effective strategies to improve awareness and knowledge regarding brucellosis, tailored to the specific needs and characteristics of each target population.

Knowledge regarding brucellosis

In this section, the participants’ knowledge was assessed, and more than half of the participants were found to have excellent knowledge of the disease. Globally, a meta-analysis of 79 observational studies revealed inadequate knowledge of brucellosis, particularly in Asia and Africa [10]. However, two studies conducted in Saudi Arabia reported good knowledge of brucellosis among their target populations [6,7]. The participants’ knowledge levels varied depending on their residency area, which poses challenges in controlling and preventing brucellosis in the Qassim region. Therefore, other areas of Qassim should receive more attention in awareness campaigns.

Participants with higher education levels demonstrated greater knowledge regarding brucellosis. In addition, similar studies revealed the same finding [6,7,11,13]. Participants’ knowledge was positively correlated with their monthly income, showing that participants with a higher monthly income have a higher level of knowledge compared with others. This finding is consistent with a study conducted in Jazan [7].

Regarding the level of knowledge and the source of information, most participants acquired their knowledge from friends. However, participants who were knowledgeable about brucellosis received information from healthcare practitioners, indicating the importance of healthcare practitioners’ involvement in brucellosis campaigns.

Attitudes and practices regarding brucellosis

Our questionnaire included a section specifically for livestock owners, in which we explored their attitudes and practices that could influence the spread of brucellosis in the community. Similar to a previous study in Saudi Arabia, we could not find a significant relationship between the level of knowledge about brucellosis and various attitudes and practices related to the disease. Our data showed that approximately three-fourths of livestock owners consume meat from the animals that they raise and nurture, which is an expected behavior among livestock owners. However, this practice is more common in the Qassim region than in Jazan [7]. Generally, no specific recommendations were made in favor of or against consuming meat from one’s own livestock. Approximately two-thirds of our sample reported participating in the slaughtering of their livestock. In contrast, among the pastoral community in Kenya, only 36% participated in slaughtering animals. However, the seroprevalence of brucellosis among livestock in Kenya is higher than in the Qassim region of Saudi Arabia [14,15].

Only 6.7% of livestock owners in our study reported always wearing gloves while caring for their livestock. According to the Food and Agriculture Organization (FAO), wearing gloves when handling livestock is a preventive measure against brucellosis transmission. In addition, 38.7% of livestock owners participated in delivering a pregnant animal with an abortion, thus exposing themselves to aborted animals. The FAO considers this a risk factor for brucellosis. In the Jazan study, data regarding wearing gloves and delivering pregnant animals are somehow similar to the data collected in the Qassim region for this study [7,16].

Regarding drinking unpasteurized milk, which is a known risk factor for brucellosis [4], approximately half of the respondents reported consuming fresh milk without boiling it. In a study conducted in Tajikistan, this practice is less common, reported by only 28% of small-scale dairy farmers in urban and peri-urban areas [11]. Regarding livestock vaccination against brucellosis, in Saudi Arabia, vaccinating livestock against brucellosis is not mandatory but recommended and provided by the Ministry of Environment, Water, and Agriculture (MEWA) [17,18]. In our study, 65.3% of the respondents reported vaccinating their livestock against brucellosis. In other countries, such as China, Argentina, and Brazil, livestock vaccination against brucellosis is mandatory [19-21]. The effectiveness of mandatory vaccinations in Brazil has positively reduced the prevalence of brucellosis [22].

We recommend that the MEWA initiate awareness campaigns on brucellosis, specifically targeting population groups with limited awareness and knowledge regarding brucellosis. Furthermore, MEWA should consider a comprehensive national program against brucellosis, which may even include mandatory livestock vaccinations.

Limitations

This study encountered a few limitations. We believe that distributing an online questionnaire rather than a self-administered questionnaire may result in misinterpretations of the questions. In addition, we employed a nonprobability sampling technique. The participants who volunteered to participate may not be representative of the entire population. Furthermore, this study utilized a cross-sectional design that solely captured data at a specific moment in time. A longitudinal study design can provide insights into changes in awareness, knowledge, and practices over time and help identify trends or evaluate the effectiveness of awareness campaigns or interventions.

## Conclusions

This study assessed the knowledge, attitudes, and practices regarding brucellosis among the general population in the Qassim region. The results demonstrated a moderate level of awareness, with the majority of participants exhibiting good knowledge about brucellosis. The study also explored attitudes and practices related to brucellosis, revealing unsafe behaviors, such as the consumption of raw milk, and inadequate preventive practices, including not wearing gloves during livestock slaughter. These findings underscore the significance of promoting safe practices, such as animal vaccination and proper food handling, to prevent the transmission of brucellosis. Notably, no significant relationship was found between the level of knowledge about brucellosis and different attitudes and practices toward the disease; future studies employing random sampling techniques are required to further prove this finding. We recommend implementing strategies to improve the level of awareness, such as campaigns, especially among younger age groups and those living in some governorates of the Qassim region. However, given the lack of association between the level of knowledge and preventive attitudes practiced by livestock owners, we believe that authorities should improve their strategies in dealing with the spread of brucellosis. This approach should not only depend on awareness campaigns but also on the implementation of a highly comprehensive plan that includes mandatory livestock vaccination against brucellosis and increased supervision of livestock slaughter sites.
